# Adenosine receptor signaling: a key to opening the blood–brain door

**DOI:** 10.1186/s12987-015-0017-7

**Published:** 2015-09-02

**Authors:** Margaret S. Bynoe, Christophe Viret, Angela Yan, Do-Geun Kim

**Affiliations:** Department of Microbiology and Immunology, College of Veterinary Medicine, Cornell University, Ithaca, NY 1853 USA; INSERM U1111-CIRI, CNRS UMR5308, Université Lyon 1 and ENS Lyon, 69365 Lyon, France

**Keywords:** Adenosine, Ectonucleotidases, P1 purinergic receptors, CNS, Brain, Blood–brain barrier, Permeability, Endothelial cells, Drug delivery

## Abstract

The aim of this review is to outline evidence that adenosine receptor (AR) activation can modulate blood–brain barrier (BBB) permeability and the implications for disease states and drug delivery. Barriers of the central nervous system (CNS) constitute a protective and regulatory interface between the CNS and the rest of the organism. Such barriers allow for the maintenance of the homeostasis of the CNS milieu. Among them, the BBB is a highly efficient permeability barrier that separates the brain micro-environment from the circulating blood. It is made up of tight junction-connected endothelial cells with specialized transporters to selectively control the passage of nutrients required for neural homeostasis and function, while preventing the entry of neurotoxic factors. The identification of cellular and molecular mechanisms involved in the development and function of CNS barriers is required for a better understanding of CNS homeostasis in both physiological and pathological settings. It has long been recognized that the endogenous purine nucleoside adenosine is a potent modulator of a large number of neurological functions. More recently, experimental studies conducted with human/mouse brain primary endothelial cells as well as with mouse models, indicate that adenosine markedly regulates BBB permeability. Extracellular adenosine, which is efficiently generated through the catabolism of ATP via the CD39/CD73 ecto-nucleotidase axis, promotes BBB permeability by signaling through A_1_ and A_2A_ ARs expressed on BBB cells. In line with this hypothesis, induction of AR signaling by selective agonists efficiently augments BBB permeability in a transient manner and promotes the entry of macromolecules into the CNS. Conversely, antagonism of AR signaling blocks the entry of inflammatory cells and soluble factors into the brain. Thus, AR modulation of the BBB appears as a system susceptible to tighten as well as to permeabilize the BBB. Collectively, these findings point to AR manipulation as a pertinent avenue of research for novel strategies aiming at efficiently delivering therapeutic drugs/cells into the CNS, or at restricting the entry of inflammatory immune cells into the brain in some diseases such as multiple sclerosis.

## Background

**Fig. 1:**
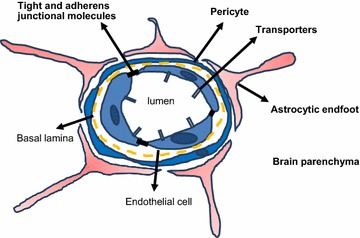
Schematic of blood brain barrier (BBB) structure and the neurovascular unit (NVU). The brain vasculature is lined with a single layer of endothelial cells that is tightly sealed by tight and adherens junction molecules. It is further insulated by pericytes and astrocytic endfoot processes and in total are referred to as the NVU. Efflux and influx transporters expressed on BBB endothelial cells selectively allow the entry or exit of molecules into or out of the brain

Neurons of the central nervous system (CNS) are separated from the lumen of blood vessels by physical barriers which ensure both protective and homeostatic functions [1, 2]. The main barriers are the blood–brain barrier (BBB) and its spinal cord counterpart, the blood-spinal cord barrier. Such barriers are made of tightly connected endothelial cells that line the CNS microvasculature and form a more highly restrictive barrier than endothelial cells of the peripheral circulation. These cells are characterized by a markedly restricted pinocytosis and trancytosis potential, the expression of dedicated transporters that regulate the influx/efflux of nutritive/toxic compounds, a low expression of leukocyte adhesion molecules and the elaboration of specialized luminal structures involved in tight and adherens junctions that efficiently restrain passive diffusion of blood-borne molecules [3–9]. The BBB endothelium is surrounded by basement membrane, pericytes and processes from neighboring astrocytes that contribute to the so-called neurovascular unit (NVU) which regulates barrier functions, homeostasis and stability [10] (Fig. 1). Astrocytes provide nutrients that are important for endothelial cells activation/polarization; and they function as a scaffold, providing structural support for the vasculature. While astrocytic processes enwrap endothelial cells, they also interact with microglial cells and neurons [11, 12]. Astrocytes regulate BBB tightness by providing soluble factors that aid in endothelial cell proliferation and growth or are involved in maintenance of BBB integrity [13, 14]. While controlling the passage of molecules between the brain blood circulation and the brain microenvironment in the healthy brain, the BBB may also contribute to the pathogenesis of several neurological disorders such as neurodegenerative diseases, under conditions of abnormal functioning [1]. Therefore, dissecting the mechanisms underlying the properties of the BBB is necessary for understanding both the physiology of the healthy CNS as well as the development of some brain pathologies.

Adenosine is a nucleoside naturally produced by neurons and glial cells. Through a well characterized set of receptors called P1 purinergic receptors, adenosine has long been known to act as a potent modulator of various brain functions through the regulation of multiple neurotransmitters, receptors and signaling pathways [15]. Here, we review recent in vivo and in vitro studies that point to the adenosine-AR axis as an important regulatory pathway controlling BBB permeability to macromolecules and cells, and propose that manipulation of AR signaling might represent a new approach to achieve an efficient delivery of therapeutic agents into brain parenchyma.

## Adenosine and ARs in CNS physiology

**Fig. 2:**
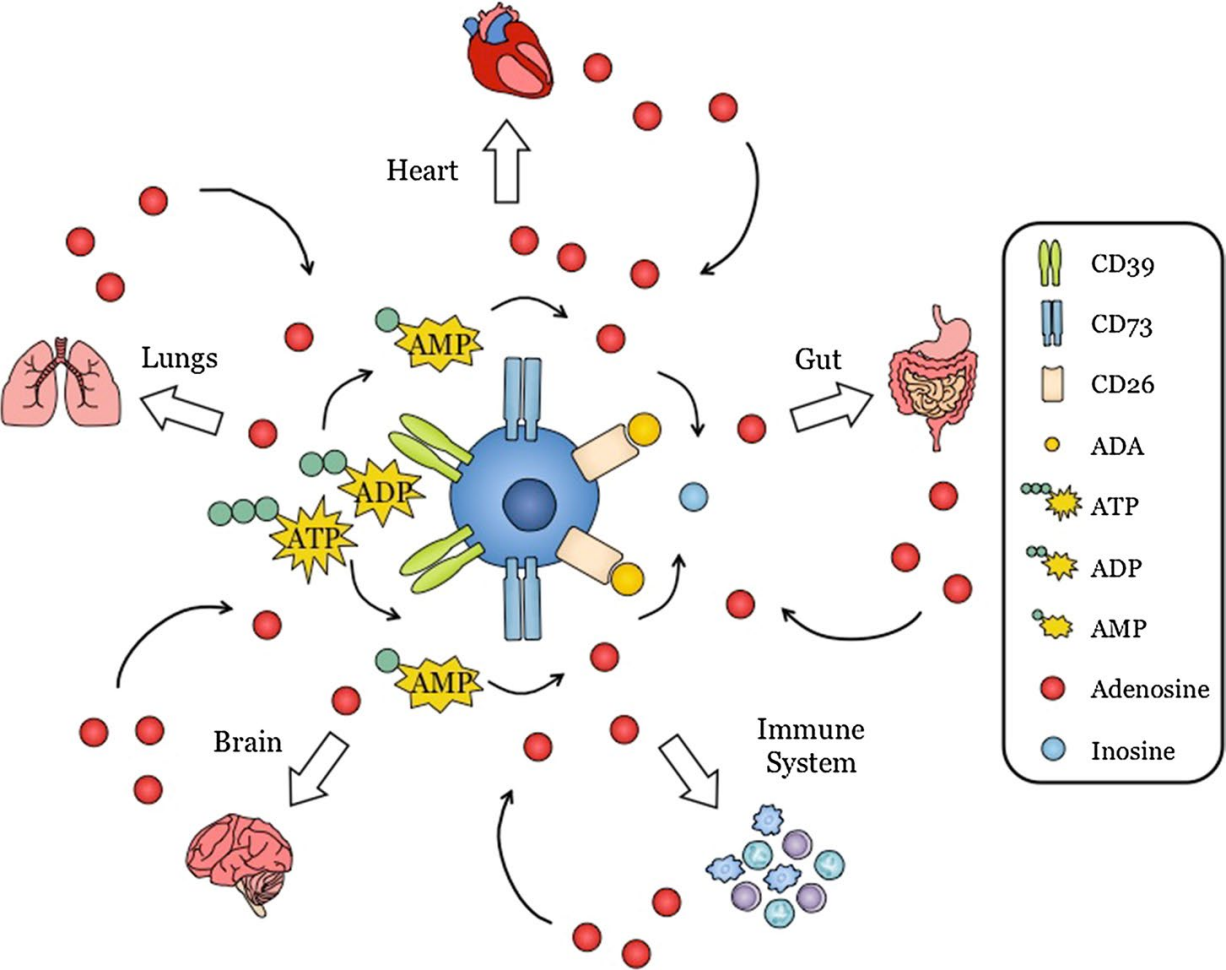
Adenosine is a purine nucleoside produced by many different organs throughout the body. Extracellular adenosine is a primordial molecule that is produced by many cell types in the body. These include heart, lung, gut, brain and immune cells. Adenosine produced by these cells can in turn act on the producing cells or on adjacent cells to modulate function. Extracellular adenosine is produced from ATP released in the extracellular environment upon cell damage and is converted to ADP and AMP by CD39. AMP is further converted to adenosine by CD73. Extracellular adenosine binds to its receptors expressed on the same cell or adjacent cells to mediate its function. Adenosine is rapidly degraded to inosine by adenosine deaminase

**Fig. 3:**
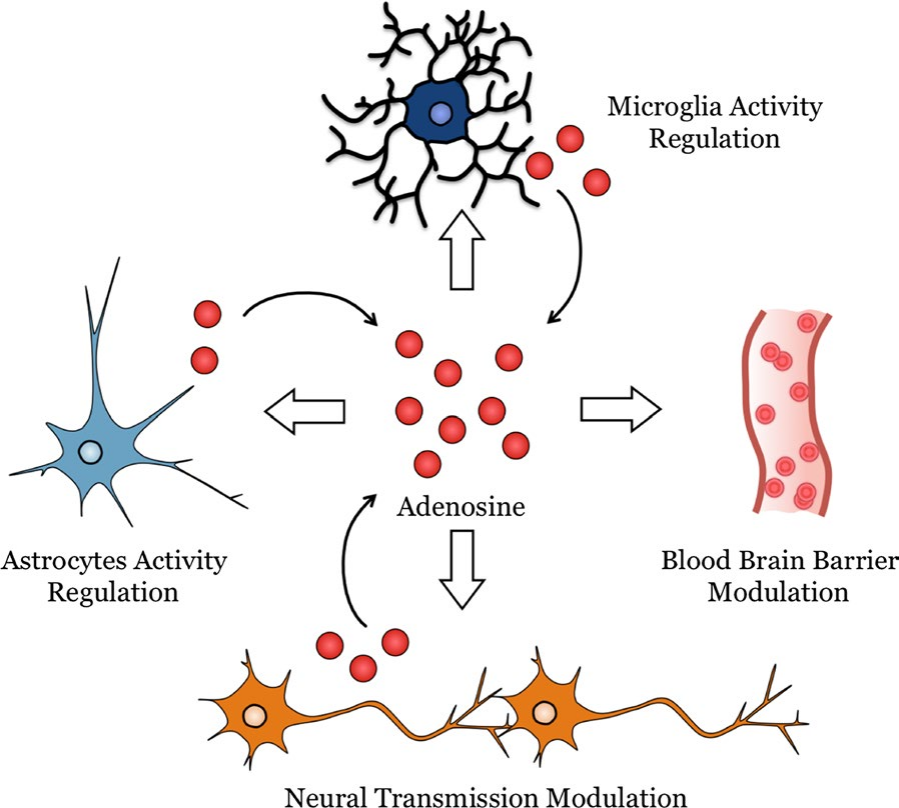
Cells of the central nervous system (CNS) not only produce adenosine but are also regulated by adenosine. Cells of the CNS, such as astrocytes, microglia, pericytes and neuronal cells can produce adenosine or their activity/function is regulated by adenosine. Adenosine regulates the blood brain barrier permeability and is involved in neural transmission and glial cell immune function and metabolism

Adenosine is a purine nucleoside involved in a myriad host functions. It is a potent immune regulator and, in addition, is notable for its role in regulating inflammation, wound healing, angiogenesis and myocardial contractility (Fig. 2). Within the CNS, adenosine is released by both neurons and glial cells. It regulates multiple physiological functions such as sleep, arousal, neuroprotection, learning and memory, cerebral blood circulation as well as pathological phenomena such as epilepsy. These effects involve adenosine modulation of neuronal excitability, vasodilatation, release of neurotransmitters, synaptic plasticity/function and local inflammatory processes [15–17] (Fig. 3).

Within cells, adenosine is an intermediate for the synthesis of nucleic acids and adenosine triphosphate (ATP). It is generated from 5′-adenosine monophosphate (AMP) by 5′-nucleotidase and can be converted back to AMP by adenosine kinase. Adenosine can also be derived from S-adenosylhomocysteine (SAH) due to the activity of SAH hydrolase. Intracellular adenosine is metabolized into inosine by adenosine deaminase (ADA) and into AMP by adenosine kinase. Inosine formed by deamination can exit the cell intact or can be degraded to hypoxanthine, xanthine and ultimately uric acid. A low level of cellular adenosine can be quickly released in the extracellular space via equilibrative nucleoside transporters (ENTs). This release increases when intracellular adenosine concentration is augmented (ischemia, hypoxia, seizures).

Importantly, adenosine can also be directly generated outside the cell through the breakdown of cell-released adenosine tri/diphosphate (ATP/ADP) by coupled cell surface molecules with catalytically active sites (ectonucleotidases) that are abundant in the brain. The ecto-nucleoside triphosphate diphosphohydrolase 1 (E-NTPDase1) or CD39, converts ATP/ADP into AMP and the glycosyl phosphatidylinositol(GPI)-linked ecto-5′-nucleotidase (Ecto5′NTase) or CD73, converts AMP to adenosine by promoting the hydrolysis of phosphate esterified at carbon 5′ of nucleotides with no activity for 2′- and 3′-monophosphates [18, 19]. Human CD73 assembles as a dimer of GPI-anchored glycosylated mature molecules. Each monomer contains an N-terminal domain that binds divalent metal ions and a C-terminal that binds the nucleotide substrate. The ectonucleotidases-mediated generation of adenosine from adenine nucleotides is very rapid (about 1 ms). Adenosine half-life in the extracellular space is about 10 s. Under basal conditions, most extracellular adenosine appears to re-enter cells through equilibrative transporters. A small fraction can be irreversibly converted into inosine and its derivatives (hypoxanthine, xanthine, uric acid) by ADA and xanthine oxidase (Fig. 2). Such a fraction increases under conditions of hypoxia/ischemia [20, 21]. Extracellular adenosine can also be targeted by ectokinases to regenerate AMP, ADP and ATP. The concentration of extracellular adenosine is maintained at low levels within the brain (ranging from 25 to 250 nM) which represent the balance between the export/generation of extracellular adenosine and its metabolism. Under pathophysiological circumstances, such as hypoxia or ischemia, extracellular adenosine concentrations can increase up to 100 fold [22, 23]. Because of its rapid metabolism, adenosine acts locally rather than systemically [24].

Extracellular adenosine exerts its action through seven-transmembrane domain, G-protein coupled receptors (GPCRs) that are connected to distinct transduction pathways. There are four different subtypes of ARs, A_1_, A_2A_, A_2B_ and A_3_ with distinct expression profiles, pharmacological characteristics and associated signaling pathways [25, 26]. A_1_ and A_3_ ARs are inhibitory and suppress adenylyl-cyclase which produces cyclic-AMP (cAMP) while A_2A_ and A_2B_ ARs are stimulatory for adenylyl-cyclase [27]. In turn, A_2B_-induced cAMP can upregulate CD73 [28]. A_1_ and A_2A_ ARs have high affinity for adenosine (about 70 and 150 nM respectively) whereas A_2B_ and A_3_ have a markedly lower affinity for adenosine (about 5100 and 6500 nM, respectively) [27]. This suggests that A_1_ and A_2A_ may be the major ARs that are activated by physiological levels of extracellular adenosine within the CNS. Accordingly, unlike A_1_ and A_2A_ receptors, A_2B_ receptor engagement in the brain is triggered by higher adenosine levels such as levels associated with cell stress or tissue damage [25]. The expression level of ARs varies depending on the type of cells or organs where they are expressed [22].

The influence of adenosine in the CNS depends both on its local concentration and on the expression level of ARs. The A_1_ receptor is highly expressed in the brain cortex, hippocampus, cerebellum and in spinal cord [25, 29, 30] and at lower levels at other sites of the brain [31]. In multiple sclerosis (MS) patients, the A_1_ receptor expression level appears to be decreased in CD45 positive glial cells of the brain [32]. The A_2A_ receptor expression is high in the olfactory tubercle, dorsal and ventral striatum and throughout the choroid plexus which forms the blood-cerebrospinal fluid (CSF) barrier [33–36] and more moderate in the meninges, cortex and hippocampus [29, 33, 36]. The steady state expression of A_2A_ receptor permits for example the proper regulation of extracellular glutamate titer by adenosine, through modulation of glutamate release and control of glutamate transporter-1-mediated glutamate uptake [37–39]. A_2A_ receptors interact negatively with D2 dopamine receptors [40]. A_2A_ receptor expression in glial cells such as astrocytes is substantially upregulated by stress factors including pressure, pro-inflammatory factors (interleukin (IL)-1β, tumor necrosis factor (TNF)-α) or hypoxia. In contrast to A_1_ and A_2A_, A_2B_ and A_3_ receptors are expressed at relatively low levels within brain [31].

## Expression of the CD73 ecto-enzyme on CNS barrier cells

**Fig. 4:**
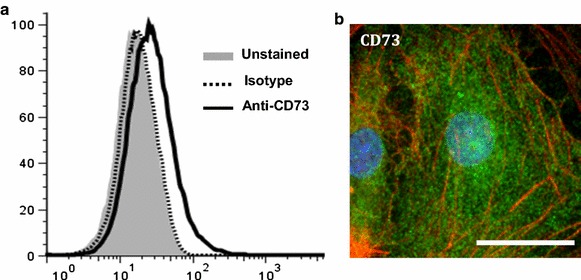
CD73 expression on primary brain endothelial cells (EC). **a** Histogram depicting CD73 expression on primary brain endothelial cells isolated from naïve, WT, C57BL/6 mice after staining with a monoclonal antibody to CD73 and analyzed by FACS. **b** Expression of CD73 (*green*) on cultured primary human brain endothelial cells visualized by immunofluorescent microscopy. Cells were counterstained with F-actin (*red*). *Scale bar* is 50 μm

Some studies have pointed to CD73 as a regulator of tissue barrier function [41]. Within the CNS, ATP can be released from neurons or other cells such as astrocytes. As mentioned above, CD39 catalyses the conversion of proinflammatory ATP/ADP into AMP and CD73 subsequently converts AMP into adenosine [42]. Thus, the proper functioning of CD39/CD73 ectonucleotidases concomitantly ensures the production of extracellular adenosine and the extinction of purinergic P2 receptor-dependent, ATP-induced signaling due to reduction of the ATP/ADP pool. Both of these effects contribute to the anti-inflammatory potential of the CD39/CD73 axis. Along with colon and kidney, the brain has particularly high levels of CD73 enzyme activity [41]. Similar to the A_2A_ receptor, CD73 shows its strongest expression level in the CNS within the choroid plexus epithelium and is also detected on glial cells of the submeningeal areas of the spinal cord [22, 27, 43]. CD73 can be expressed on many types of endothelial cells [44]. Its expression on BBB endothelial cells remains low under steady state conditions relative to peripheral endothelial cells (Fig. 4a). It is present on mouse (Bend.3) and human (hCMEC/D3) brain endothelial cell lines in vitro [27, 45]. Unlike human brain endothelial cells [46, 47], CD73 expression on primary mouse brain endothelial cells was very low and not detected in vivo [43] (Fig. 4a). However, CD73 expression can be detected in primary human brain endothelial cells (Fig. 4b) [45, 48]. CD73 expression is sensitive to cyclic AMP (cAMP) and hypoxia-inducible factor (HIF)1 through its promoter [49]. Interferon (IFN)-β increases CD73 expression and adenosine concentration at the level of the CNS microvasculature, BBB and astrocytes [46, 47] and through enhanced adenosine production, may contribute to the anti-inflammatory effect of IFN-β in MS treatment.

## AR signaling in the NVU

**Fig. 5:**
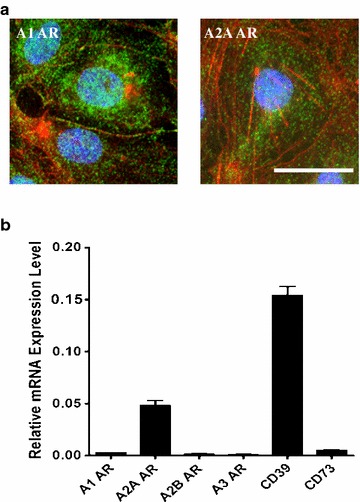
Expression of adenosine receptors (ARs) and adenosine-generating enzymes on brain endothelial cells. **a** A_1_ and A_2A_ ARs expression *green* on primary human brain endothelial cells by immunofluorescence assay (IFA). Cells were counterstained with F-actin (*red*). **b** Relative mRNA expression level of ARs, CD39 and CD73 by quantitative PCR (q-PCR) in mouse primary brain endothelial cells. *Scale bar* is 50 μm

Functional A_1_, A_2A_, A_2B_ and A_3_ receptors are all expressed at moderate levels in glial cells under physiological context [50] and this level is upregulated under inflammatory conditions or brain injury. All P1 purinergic receptors appear to be present on cultured oligodendrocytes [51], on microglial cells [42] and are functional on astrocytes [52–56] (Fig. 3). In astrocytes, ARs engagement is not only important for glutamate uptake regulation (A_2A_ receptor) but also to maintain cellular integrity (A_1_ receptor) [53, 57, 58], protect from hypoxia-related cell death (A_3_ receptor) [57] and regulate CCL2 chemokine production (A_3_ receptor) [59]. In microglial cells, A_2A_ receptor engagement inhibits process extension and migration while A_1_ and A_3_ receptors engagement have the opposite effect [42]. A_1_ and A_2A_ receptor transcripts are detectable in Z310 epithelial cells derived from mouse choroid plexus [43]. As to CNS endothelial cells, A_1_, A_2A_ and A_2B_ receptor transcripts and proteins were expressed in hCMEC/D3 human brain endothelial cells [45]. Also, A_1_ and A_2A_ ARs are expressed in primary human brain endothelial cells (Fig. 5a). In Bend.3 mouse brain endothelial cells, transcripts and proteins for A_1_ and A_2A_ receptors were detected [27]. Finally, both A_1_ and A_2A_ receptor proteins were found expressed in primary mouse brain endothelial cells and transcripts and proteins for both A_1_ and A_2A_ receptors were present in brain endothelial cells in mice [27] (Fig. 5b).

## AR signaling and CNS barrier permeability

The recent notion that adenosine could play a substantial regulatory role in CNS barrier permeability stems from the observation that extracellularly generated adenosine positively regulates the entry of lymphocytes into the brain and spinal cord during disease development in the experimental autoimmune encephalomyelitis (EAE) model [43] and the observation that irradiated A_2A_ AR deficient mice reconstituted with wild-type bone marrow cells developed only very mild signs of EAE with virtually no CD4^+^ T cell infiltration in spinal cord [43]. In line with an important role for AR signaling in regulating the permeability of the BBB is the observation that inhibition of ARs by caffeine (a broad-spectrum AR antagonist) prevents the alteration of BBB function induced by cholesterol or 1-methyl-4-phenyl-1,2,3,6-tetrahydropyridine (MPTP) in animal models of neurodegenerative diseases [60, 61].

Recent observations support the notion that engagement of ARs on brain endothelial cells modulates BBB permeability in vivo. Experimental recruitment of ARs either by the broad spectrum agonist NECA or the engagement of both A_1_ and A_2A_ receptors by selective agonists (CCPA and CGS21680) cumulatively and transiently augmented BBB permeability facilitating the entry of intravenously infused macromolecules (including immunoglobulins such as the anti-β-amyloid 6E10 antibody) into the CNS [27]. Accordingly, the analysis of engineered mice lacking these receptors reveals a limited entry of macromolecules into the brain upon exposure to AR agonists. CNS entry of intravenously delivered macromolecules was also induced by the FDA-approved, A_2A_ AR agonist Lexiscan: 10 kDa dextran was detectable within the CNS of mice as soon as 5 min after drug injection. The maximum increase in BBB permeability was observed at about 30 min after injection both in mice and rats. The limited half-life of Lexiscan (about 3 min) is likely to account for the lower duration of BBB permeability relative to that induced by NECA (half-life: 5 h).

Upon exposure to NECA or Lexiscan, monolayers of Bend3 mouse brain endothelial cells (CD73^+^, A_1_ AR^+^, A_2A_ AR^+^) lowered their transendothelial cell electrical resistance, a phenomenon known to be associated with increased paracellular space and augmented permeability [62, 63]. AR activation by agonists was indeed associated with augmented actinomyosin stress fiber formation indicating that ARs signaling initiates changes in cytoskeletal organization and cell shape. These processes are reversed as the half-life of the AR agonist decreases. At the level of tight junctions, signaling induced by A_1_ and A_2A_ receptor agonists altered the expression level of tight junction proteins such as claudin-5 and ZO-1, and particularly of occludin in cultured brain endothelial cells [27]. The exact signaling circuits connecting AR engagement and cytoskeletal remodeling remain to be dissected. In agreement with these findings and with the observation that human brain endothelial cells do respond to adenosine in vitro, agonist-induced A_2A_ receptor signaling transiently permeabilized a primary human brain endothelial cell monolayer to the passage of both drugs and Jurkat human T cells in vitro [48]. Interestingly, transendothelial migration of Jurkat cells was primarily of the paracellular type. The permeabilization process involved RhoA signaling-dependent morphological changes in actin-cytoskeletal organization, a reduced phosphorylation of factors involved in focal adhesion (namely Ezrin-Radixin-Moesin (ERM) and focal adhesion kinase (FAK)) as well as a marked downregulation of both claudin-5 and vascular endothelial (VE)-cadherin [48], two factors instrumental for the integrity of endothelial barriers. Hence, by regulating the expression level of factors crucially involved in tight junction integrity/function, signaling induced through receptors for adenosine acts as a potent, endogenous modulator of BBB permeability in mouse models as well as in human cellular models in vitro.

Some G proteins such as G_α_ subunits can influence the activity of small GTPases RhoA and Rac1 that are known modulators of cytoskeletal organization. RhoA and Rac1 are responsive to adenosine signaling and promote actin cytoskeleton remodeling [62, 64–66]. The precise molecular events linking A_1_/A_2A_ AR engagement to changes in the expression pattern of factors involved in tight junction functioning remains however to be analyzed in detail. In particular, whether both canonical (G protein-dependent) and non-canonical (e.g. G protein-independent, β-arrestin-related) signaling pathways contribute to such regulation is an open question. Another interesting issue relates to the capacity of A_1_ and A_2A_ receptors to form heterodimers [67] and the possible impact of such oligomeric receptors on the regulation of CNS barrier by ARs agonists.

## CD73 and AR signalling in immune cell entry into the CNS

Besides its capacity to regulate the local inflammatory context through consumption of ATP and generation of adenosine, the expression of CD39/CD73 by endothelial cells can regulate homeostasis by preventing high local concentrations of ATP that promote thrombosis and generating adenosine which instead, contributes to an antithrombotic microenvironment [44]. The CD39/CD73 axis also regulates leukocyte migration induced by chemokines [68, 69] and immune cell adhesion to endothelial cells. Such adhesion is favored by high ATP concentrations and limited by adenosine, with mutant mice lacking CD39 or CD73 having augmented level of leukocyte adhesion to endothelial cells [70, 71]. Thus, adenosine contributes to restraining leukocyte recruitment and platelet aggregation and might be important to control vascular inflammation.

We have observed that CD73-generated adenosine promotes the entry of inflammatory lymphocytes into the CNS during EAE development [43]. Genetically manipulated mice unable to generate extracellular adenosine due to deficiency in the ectonucleotidase CD73 (CD73^−/−^) are resistant to lymphocyte entry into the CNS and EAE development relative to wild type animals and such a phenotype could be recapitulated in regular mice by using either the broad spectrum AR antagonist, caffeine or the SCH58261 that selectively antagonizes the signaling induced by adenosine-bound A_2A_ receptor [43, 72–74]. This effect was remarkable since auto-reactive lymphocytes from CD73^−/−^ mice indeed harbor an enhanced inflammatory potential. In addition, the expression of CD73 (and presumably its enzymatic activity) on either T cells or CNS cells was sufficient to support lymphocyte entry into the CNS since CD4 T cells from wild type donors (i.e. CD73^+^) could mediate a milder yet substantial level of EAE pathogenesis in CD73^−/−^ recipient mice [43].

Since CD73 and the A_1_/A_2A_ receptors are expressed at the level of the choroid plexus, locally produced extracellular adenosine is likely to act in an autocrine manner. Given that A_1_ and A_2A_ receptor recruitment are functionally opposed to each other and harbor some differences in their affinity for adenosine [26], the regional extracellular concentration of adenosine may strongly influence the response of neighboring cells expressing both receptors. A_1_ receptor signaling may be involved at low adenosine concentrations while A_2A_ receptor signaling is likely to become prominent at elevated adenosine concentrations. Thus, CD73 enzymatic activity at the choroid plexus and the regional adenosine levels are likely to influence local inflammatory events. Interestingly, the choroid plexus is suspected to represent a primary entry site for immune cells during neuroinflammation [3–5] and for steady state immunosurveillance [6, 75]. By combining the gene expression pattern of chemokines and chemokine receptors relevant to EAE in CD73 null mutant versus control mice developing EAE and the effect of the broad spectrum AR agonist NECA on the expression profile of these molecules in unmanipulated animals, it was possible to identify CX3CL1/fractalkine, a chemokine/adhesion molecule [76], as the major factor induced by extracellular adenosine in the brain of mice developing EAE [77]. The cleavage of the cell surface-expressed form of CX3CL1 by ADAM-10 and −17 factors generates a local CX3CL1 gradient [78]. The selective A_2A_ AR agonist CGS21680 caused an increase in CX3CL1 level in the brain of treated mice. Conversely, the A_2A_ AR antagonist SCH58261 protected mice from CNS lymphocyte infiltration and EAE induction recapitulating the phenotype of CD73 null mutant mice. Thus, the augmented CX3CL1 expression level seen in the brain of EAE developing mice can be regulated by A_2A_ AR signaling. During EAE, the greatest increase in CX3CL1 occurred at the choroid plexus and returned to normal when mice recovered from disease. As choroid plexus cells express both CD73 and A_2A_ AR, they have the intrinsic capacity to generate and respond to extracellular adenosine. In vitro, A_2A_ AR engagement on the choroid plexus epithelial cell line CPLacZ-2 by CGS21680 induced CX3CL1 expression and promoted lymphocyte transmigration suggesting that CX3CL1 induction by extracellular adenosine contributes to lymphocyte migration into the brain parenchyma during EAE. In agreement with an important role for CX3CL1 in EAE pathogenesis is the fact that CX3CL1 blockade by neutralizing antibodies prevented lymphocyte entry into the CNS and EAE development [77]. This notion is in line with the elevated serum level of CX3CL1 which can be observed during CNS inflammation including MS patient brain lesions [79–81]. Importantly, there was a positive correlation between CX3CL1 expression levels and the relative frequency of lymphocytes present in the CSF of inflamed brains [80, 82]. Moreover, relative to BBB endothelial cells, choroid plexus epithelial cells constitutively express high levels of CD73. Blockade of CD73 or A_2A_ AR inhibits inflammatory cells entry into the CNS [43, 77]. Thus, at the level of the choroid plexus, induction of A_2A_ receptor signaling by elevated local adenosine concentrations is likely to contribute to immune cell entry into the brain parenchyma.

Among immune cells, the CX3CL1 receptor (CX3CR1) was detected on a sizable fraction of CD4 T cells, CD8 T cells, macrophages and NK cells in mice [33]. CNS CX3CL1 might also modulate neuroinflammation by recruiting a subset of CNS resident NK cells able to attenuate the aggressiveness of autoreactive CD4 T cells of the Th17 effector type [83, 84]. Interestingly, while the frequency of inflammatory immune cells is significantly decreased in the CNS of A_2A_ AR^−/−^, CD73^−/−^ or mice treated with A_2A_ AR antagonist, the numbers/frequency of CD4^+^CD25^+^ T regulatory cells in these mice were similar to WT. This suggests that CD73/A_2A_ AR signaling may preferentially regulate inflammatory immune cells entry into the CNS but confers less stringency on these suppressor T cells.

## Perspectives for improved delivery of therapeutic factors within the CNS

Although the BBB serves a protective role, it can constitute a complication for treatment in CNS diseases by hindering the entry of therapeutic compounds into the brain [85]. Researchers have focused on uncovering ways to manipulate the BBB to promote access to the CNS [86]. Determining how to safely and effectively do this could impact the treatment of various neurological diseases, ranging from neurodegenerative disorders to brain tumors. This implies simultaneous treatments with agents susceptible to increase permeation of CNS barriers. Current approaches involve barrier disruption which is induced by drugs such as mannitol or Cereport/RMP-7. Hypertonic mannitol, is active through shrinking of endothelial cells [87, 88] but can cause epileptic seizures and does not allow for repeated use [89, 90]. The bradykinin analog (Cereport/RMP-7) has shown some potential in transiently increasing normal BBB permeability [91] but did not give satisfactory results in clinical trial [92] despite some efficacy in treating rodent models of CNS pathologies [93–96].

The barrier permeability can also be circumvented, for instance by direct injection of drugs into ventricles [87, 97]. More recent approaches involve the delivery of drugs during compression waves induced by high-intensity focused ultrasounds [98]. Both these approaches are invasive and may lead to permanent brain damage. Another strategy involves chemical modifications of compounds in order to confer upon them some capacity to cross CNS barriers. For example, increasing the lipophilicity of drugs can enhance their capacity to cross the BBB although it often requires an increase in their size which limits the cell penetration capacity [99]. Alternatively, therapeutic compounds can be linked to factors that trigger receptor-mediated endocytosis. Coupling a compound to an antibody directed to the transferrin receptor can promote delivery of proteins to the brain in rats [100, 101]. However, the endocytic activity of endothelial cells is rather limited at the BBB and the expression level of the relevant receptor needs to be sufficient.

**Fig. 6:**
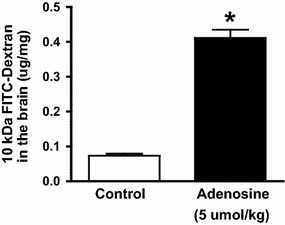
Adenosine increases the permeability of the blood brain barrier to 10 kDa FITC dextran. Concomitant administration of Adenosine and 10 kDa FITC-Dextran in C57BL/6 mice with exogenous adenosine induces significantly higher accumulation of FITC-Dextran into the brain than PBS control treatment group (n = 2, *asterisk* indicates p < 0.01)

For adenosine to exert biological effect, CD73 and ARs must be present on the same cell or on adjacent cells, because adenosine acts locally due to its short half-life. CD73, A_1_ and A_2A_ ARs are indeed expressed on BBB endothelial cells in mice and humans. While CD73 is highly and constitutively expressed on choroid plexus epithelial cells that form the blood to CSF barrier, its expression on brain endothelial barrier cells is low under steady state conditions, but increases in neuroinflammatory diseases or under conditions where adenosine is produced in response to cell stress/inflammation or tissue damage. In mice, pharmacological activation or inhibition of the A_2A_ AR expressed on BBB cells opens and tightens the BBB, respectively, to entry of macromolecules or cells. The observation that adenosine can modulate BBB permeability upon A_2A_ receptor activation suggest that this pathway might represent a valuable strategy for modulating BBB permeability and promote drug delivery within the CNS [27, 48]. Factors such as the FDA-approved, A_2A_ AR agonist Lexiscan, or a broad-spectrum agonist, NECA, increased BBB permeability and supported macromolecule delivery to the CNS in experimental setting [27]. Such exogenous agonists might represent a new avenue of research for therapeutic macromolecule delivery to the human CNS. Of note is the fact that the window of the induced permeability correlated with the half-life of the agonist. Thus, BBB permeation induced by NECA treatment (half-life, 4 h) lasted significantly longer than that induced by Lexiscan treatment (half-life, 2.5 min) [27]. Interestingly, despite its short half-life, extracellular adenosine permeabilized the BBB to entry of 10 kDa dextran (Fig. 6). Approaches based on the use of such an agonist might be useful for the delivery of therapeutic antibodies to the CNS since invasive delivery is a commonly used method [102] and is not patient-friendly.

**Fig. 7:**
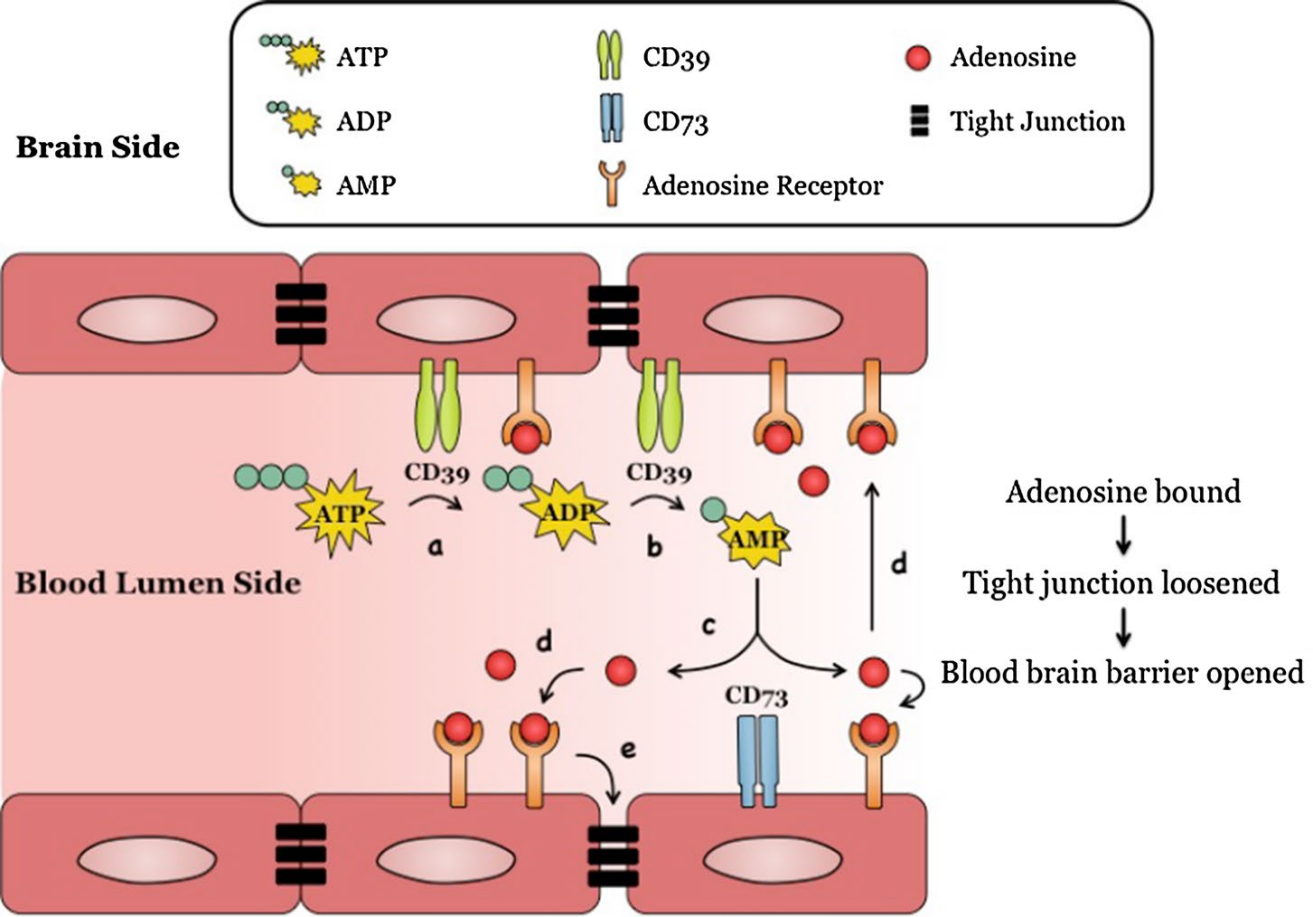
A model: Adenosine modulation of blood brain barrier (BBB) permeability. Endothelial cells lining the brain vasculature express adenosine receptors (ARs) and CD39 and CD73. In the presence of cell stress/inflammation or tissue damage (**a**), ATP is released and is rapidly converted to ADP and AMP by CD39 (**b**) and AMP is converted to adenosine by CD73. **c** Adenosine binds to its receptor/s (A_1_ or A_2A_) on BBB endothelial cells (**d**), the activation of which induces reorganization of actin cytoskeleton in BBB endothelial cells, resulting in tight and adherens junction disassembly (**e**), increasing paracellular permeability

Further studies are needed to better understand the mechanisms involved in BBB permeability modulation by A_1_/A_2A_ receptors-triggered signaling as well as the parameters susceptible to optimize the timing of such a modulation (Fig. 7). In particular, in vitro cell based model of BBB where cerebral endothelial cells are co-cultured with other components of the NVU such as pericytes or astrocytes (co-cultures and triple co-cultures systems) [103] should be considered for evaluation. Another important issue relates to the question of the identification of the CNS areas where the microvasculature is significantly permeabilized by A_1_/A_2A_ receptor-induced signaling and more generally whether or not, there exists a restricted or a global permeabilization within the CNS. An alternative strategy to be explored is the experimental manipulation of the regional level of endogenous adenosine or of the responsiveness/expression level of A_1_/A_2A_ receptors. Such knowledge will be instrumental in designing novel approaches for the improved delivery of drugs, therapeutic monoclonal antibodies and possibly, stem cells, within the CNS.

## Concluding remarks

Inhibiting AR signaling on BBB cells restricts the entry of macromolecules and inflammatory immune cells into the CNS with limited impact on anti-inflammatory, T regulatory cells. Conversely, activation of ARs on BBB cells promotes entry of small molecules and macromolecules in the CNS in a time-dependent manner. The duration of BBB permeabilization depends on the half-life of the AR activating agent or agonist, suggesting that AR modulation of the BBB is a tunable system. We conclude that: (1) The adenosine-based control of the BBB is an endogenous mechanism able to regulate cells and molecules entry into the CNS in basal conditions and during response to CNS stress or injury. (2) AR-induced opening of the BBB is time-dependent and reversible. (3) A tight regulation of CD73 expression on BBB cells is crucial to restrict and regulate adenosine bioavailability and prevent promiscuous BBB permeability.

Consequently, the control of BBB permeability via modulation of AR signaling is pertinent for research on the delivery of therapeutics to the CNS: (1) AR signaling is an endogenous mechanism for BBB control. (2) It has the potential for precise time-dependent control of BBB permeability. (3) Change in BBB permeability is reversible. (4) ARs are accessible directly on BBB endothelial cells. (5) Over 50 commercial reagents targeting ARs are available, with some approved by the FDA for clinical use. (6) In vivo and in vitro model systems can help to gain molecular mechanistic understanding of how adenosine naturally regulates changes in BBB permeability. Therapies aimed at treating neuro-inflammatory diseases such as MS, where inflammatory cells penetration of the CNS causes irreparable damage to CNS tissue, would ideally include one that could inhibit the entry of inflammatory immune cells into the CNS parenchyma. Many other diseases associated with CNS inflammation such as meningitis, encephalitis, and cerebritis could all benefit from inhibiting immune cell entry into the CNS. The challenge is determining how to safely and effectively do this. We hypothesize that manipulating the adenosine-ARs axis on CNS barrier cells may represent an efficient way to modulate the entry of immune cells into the CNS and to limit CNS inflammation and pathology.

## Abbreviations

ATP: adenosine tri-phosphate
AR: adenosine receptor
BBB: blood–brain barrier
BM: basement membrane
cAMP: cyclic adenosime monophosphate
CNS: central nervous system
CSF: cerebrospinal fluid
EAE: experimental autoimmune encephalomyelitis
Ecto5′NTase: ecto-5′-nucleotidase (CD73)
E-NTPDase1: Ecto-nucleoside triphosphate diphosphohydrolase 1 (CD39)
ERM: Ezrin-Radixin-Moesin
FAK: focal adhesion kinase
GPCR: G-protein coupled receptor
IL: interleukin
IFN: interferon
JAM: junction adhesion molecules
MS: multiple sclerosis
NVU: neurovascular unit
PDGF: Platelet derived growth factor
TGF: transforming growth factor
TNF: tumor necrosis factor
VEGF: vascular endothelial growth factor
VE-cadherin: vascular endothelial-cadherin
VSMC: vascular smooth muscle cells
ZO: zona occludens
